# Improved short and long term survival associated with percutaneous coronary intervention in the elderly patients with acute coronary syndrome

**DOI:** 10.1186/s12877-018-0818-z

**Published:** 2018-06-07

**Authors:** Xiaojing Chen, Salim Bary Barywani, Runa Sigurjonsdottir, Michael Fu

**Affiliations:** 1 Department of Molecular and Clinical Medicine, Institute of Medicine, Sahlgrenska Academy, University of Gothenburg, Gothenburg, Sweden; 2 Department of Cardiology, West China Hospital, Sichuan University, Chengdu, Sichuan China; 3 Section of Cardiology, Department of Medicine, Sahlgrenska University Hospital/Östra Hospital, 416 50 Göteborg, SE Sweden

**Keywords:** Acute coronary syndrome, Percutaneous coronary intervention, Elderly, Short and long outcome

## Abstract

**Background:**

Percutaneous coronary intervention (PCI) are increasingly used in daily clinical practice in elderly patients with acute coronary syndrome (ACS) despite limited evidence. The purpose of this study was to assess the impact of PCI on short and long term survivals in a large cohort of elderly patients with ACS from a “real world”.

**Methods:**

We enrolled 491 patients aged ≥70 years admitted to our institution with ACS from 2006 to 2012. Effect of PCI on short and long term survival was evaluated in both overall and a propensity score-matched cohort.

**Results:**

The mean age of the overall cohort is 83 ± 6 years. Among them, 285 were treated with PCI, whereas 206 were not. Patients treated with PCI were younger (82 ± 5 vs. 85 ± 6), more males (67% vs. 46%), with lower heart rate (77 ± 22 vs. 84 ± 21), higher eGFR (58 ± 20 vs. 47 ± 23), and less with heart failure (29% vs. 15%) (all *p* < 0.001). In both overall and propensity-matched population, improved survival was associated with PCI-treatment at 1 and 3 years (*p* < 0.001 for all comparisons). Furthermore, by using multivariate Cox proportional-hazards regression model following factors were identified as independent predictors of 3-year all-cause mortality: age (HR 1.08, 95% CI 1.00–1.16), heart rate (HR 1.02, 95% CI 1.01–1.03), eGFR (HR 3.07, 95% CI 1.63–5.77), malignancy (HR 2.03, 95% CI 1.27–4.57), prior CABG (HR 2.033, 95% CI 1.27–4.57), medication with statin (HR 0.40, 95% CI 0.19–0.86) in PCI group, whereas age (HR 1.08, 95% CI 1.03–1.13), heart rate (HR 1.01, 95% CI 1.01–1.02), hypertension (HR 1.87, 95% CI 1.01–3.49) and using of ACEI/ARB (HR 0.46, 95% CI 0.28–0.76) in non-PCI group.

**Conclusions:**

In elderly ACS patients, PCI-treatment was associated with improved 1 and 3-year survival and PCI-treated patients had different prognostic profile compared to those without PCI treatment.

## Background

The general population is gradually ageing worldwide, and cardiovascular diseases are still leading cause of morbidity and mortality in the elderly people. Advanced age is associated with an increased incidence of acute coronary syndromes (ACS) requiring urgent angiography [1]. The current ACS guidelines do no distinguish between elderly and younger patients [2–4] despite studies of percutaneous coronary intervention (PCI) in elderly population are limited. As a matter of fact, elderly adults have been largely underrepresented or excluded from most of the randomized controlled trials assessing an invasive versus conservative approach in ACS [5, 6]. Therefore, in daily clinical practice, interventional cardiologists are often reluctant to undertake PCI in very elderly individuals due to the perception of poor outcome owing to the high prevalence of associated comorbidities [2, 7–9]. However, during recent years, there were studies from registries or hospital cohort that indicated beneficial effect of PCI on outcome in octogenarians presenting with ACS [10–13]. Recently, the 1st randomized trial in elderly patients with non-ST segment elevation myocardial infarction (NSTEMI) and unstable angina pectoris demonstrated that an invasive strategy with PCI or coronary artery bypass grafting (CABG) is superior to a conservative strategy in the reduction of composite events [14]. However, this study was underpowered to assess survival. The purpose of the present study was to assess any associations between PCI and all-cause mortality (both short and long term) in a large cohort of elderly patients with ACS from a “real world”, and explore the predictors of 3-year all-cause mortality in patients treated with different strategies.

## Methods

### Study population

This study consecutively included 491 patients aged ≥70 years and suffered from acute ACS at Sahlgrenska University Hospitals/Sahlgrenska, and Sahlgrenska University Hospitals /Östra affiliated with the University of Gothenburg form 2006 to 2012. Patients came directly from ambulance or were referred from cardiology and medical departments. ACS was classified as ST segment elevation myocardial infarction (STEMI), non-ST segment elevation myocardial infarction (NSTMI) or unstable angina (UA). Criteria for STEMI were ischemic symptoms lasting> 10 min and ST elevation in two contiguous leads or new left bundle branch block. NSTEMI and UA were defined by the presence of ST segment depression or T-wave abnormalities or ischemic symptoms with (NSTEMI) or without (UA) elevation of cardiac enzyme levels above the reference range. Treatment strategy was made at discretion of clinical decision by responsible cardiologist. All PCIs were performed at a joint PCI center for both hospitals. The study protocol was approved by the Human Ethical Committee at University of Gothenburg.

### Data collection

The baseline demographic, medical history, clinical characteristics, comorbidities and medications were based on patients’ electronic medical records and entered into a database. The Cockcroft-Gault formula was used to calculate the estimated glomerular filtration rate (eGFR) to assess renal function.

### Follow up and end-points

All patients were followed from index hospitalization due to ACS for 3 years. The outcome measures for this study was 30-days, 1 and 3-year all-cause mortality. Mortality data during follow-up were obtained from the Death Registry of the National Board of Health and Welfare in Sweden.

### Statistical analysis

Categorical variables are described as percentages and compared using chi-square test or Fisher exact test as appropriated. Continuous variables were described as mean ± SD and compared using independent sample test. Cox proportional-hazard regression models were used to asses possible association between PCI and mortality, hazard ratios (HRs) and 95% confidence intervals (95% CIs) were presented. Estimates of the odd ratios (OR) and associated 95% confidence intervals (CI)were obtained from logistic regression models to identify factors associated with the usage of primary PCI.

To adjust for the bias inherent in treatment assignment, propensity score matching analysis with 1:1 nearest neighbor matching was employed. The propensity score is the propensity from 0 to 1 receive PCI treatment, given a set of know variables, and is used to adjust for potential selection bias, confounding and differences between the two group in observational studies [15]. Variables used in developing the propensity score are presented in Table 1. After propensity score matching, the final cohort consisted of 296 matched patients, 148 in the PCI-treated and 148 in non-PCI-treated groups. Both the overall cohort and the PS-matched cohort were further analyzed by Cox proportional-hazard regression models.

**Table 1:** Demographic and clinical characteristics of all study patients

Variables	Overall cohort			Matched cohort		
	Non-PCI(*n* = 206)	PCI(*n* = 285)	*P*-value	Non-PCI(*n* = 148)	PCI(*n* = 148)	*P*-value
Demographics						
Age, year	85.4 ± 6.0	81.6 ± 4.7	< 0.001	83.4 ± 5.6	82.6 ± 4.6	0.143
Gender, male	95(46.1)	189(66.3)	< 0.001	85(57.4)	87(58.7)	0.906
Weight, kg	71.6 ± 14.3	74.3 ± 13.0	0.041	72.4 ± 14.6	73.6 ± 13.0	0.472
Height, cm	169.5 ± 9.9	171.3 ± 9.5	0.066	169.4 ± 9.0	170.7 ± 9.9	0.222
BMI, kg/m^2^	24.6 ± 3.9	25.2 ± 3.8	0.096	25.0 ± 3.8	25.4 ± 4.0	0.387
Smoking	71(34.5)	136(47.7)	0.001	47(31.7)	68(45.9)	0.017
Clinical Characteristics						
STEMI	26(12.6)	154(54.0)	< 0.001	20(13.5)	76(51.3)	< 0.001
UNSTEMI	164(78.9)	95(35.4)	< 0.001	115(77.8)	59(39.9)	< 0.001
Unstable angina pectoris	17(8.3)	35(12.2)	0.181	14(9.5)	12(8.1)	0.838
Heart rate,bpm	83.9 ± 21.3	77.4 ± 22.1	0.001	81.9 ± 22.6	79.0 ± 20.6	0.247
Systolic BP, mmHg	144.4 ± 27.4	148.8 ± 26.3	0.083	145.6 ± 26.2	148.6 ± 26.6	0.332
Diastolic BP, mmHg	82.6 ± 15.4	84.3 ± 16.1	0.242	83.7 ± 15.3	84.1 ± 14.3	0.848
Laboratory findings						
Hemoglobin, g/L	128.7 ± 18.6	133.7 ± 15.9	0.003	130.1 ± 18.4	133.1 ± 15.7	0.128
eGFR, ml/min/1.73m^2^	47.2 ± 23.1	58.1 ± 20.1	< 0.001	52.2 ± 23.7	56.5 ± 19.9	0.096
Creatinine, umol/L	119.3 ± 89.8	100.2 ± 56.2	0.005	112.7 ± 94.6	99.3 ± 36.8	0.108
Comobidities						
Atrial fibrillation	46(22.3)	49(17.2)	0.166	34(23.0)	32(21.6)	0.889
History of heart failure	60(29.1)	44(15.4)	< 0.001	33(22.3)	35(23.6)	0.890
Hypertension	166(80.5)	149(52.3)	< 0.001	108(73.0)	99(66.9)	0.311
Diabetes	40(19.4)	55(19.3)	1.000	31(20.9)	35(23.6)	0.675
Hyperlipidaemia	36(17.5)	40(14.1)	0.376	27(18.2)	17(11.5)	0.141
Kidney disease	34(16.5)	51(17.9)	0.718	27(18.2)	24(16.2)	0.758
Pulmonary disease	31(15.0)	45(15.7)	0.900	20(13.5)	23(15.5)	0.742
Peripheral vascular disease	23(11.2)	20(7.0)	0.145	14(9.5)	15(10.1)	1.000
Anemia	25(12.1)	22(7.8)	0.120	13(9.7)	8(5.4)	0.366
Malignancies	15(7.3)	36(12.6)	0.071	13(8.9)	19(13.8)	0.349
Stroke	51(24.8)	30(10.5)	< 0.001	24(26.2)	23(15.5)	1.000
Prior CABG	24(11.6)	22(7.7)	0.159	17(11.5)	11(7.4)	0.321
Prior PCI	24(11.7)	34(11.9)	1.000	19(12.8)	12(8.1)	0.254
Prior valvar surgery	6(2.9)	2(0.7)	0.074	4(2.7	1(0.7)	0.371
Pacemaker	14(6.8)	15(5.3)	0.562	8(5.4)	9(6.1)	1.000
Medications						
β- Blockers	128(62.1)	187(65.6)	0.446	99(66.9)	109(73.6)	0.252
Aspirin,	145(70.4)	188(65.9)	0.328	107(72.3)	104(70.3)	0.797
ACEI/ARB	75(36.4)	137(48.1)	0.013	63(42.6)	76(51.3)	0.162
diuretics,	101(49.1)	80(28.1)	< 0.001	62(41.8)	54(36.5)	0.405
Spironolactone	19(9.2)	21(7.4)	0.505	11(7.4)	17(11.5)	0.312
Statins	60(29.1)	141(49.5)	< 0.001	54(36.5)	68(45.9)	0.125
Digoxin	18(8.7)	18 (6.3)	0.381	14(9.5)	14(9.5)	1.000

*BMI* body mass index, *ADL* activities of daily living, *LV* left ventricular, *LA* left atrial, *RA*, right atrial, *MR*, mitral regurgitation, *AS* aortic valve stenosis, *TR* tricuspid regurgitation, *ACEI* angiotensin converting enzyme inhibitor, *ARB* angiotensin receptor blocker

A *p* value of < 0.05 was considered statistically significant. All statistical analyses were performed using SPSS 22 statistical software (SPSS, Inc., Chicago, IllInos).

## Results

### Clinical characteristics

**Fig. 1:**
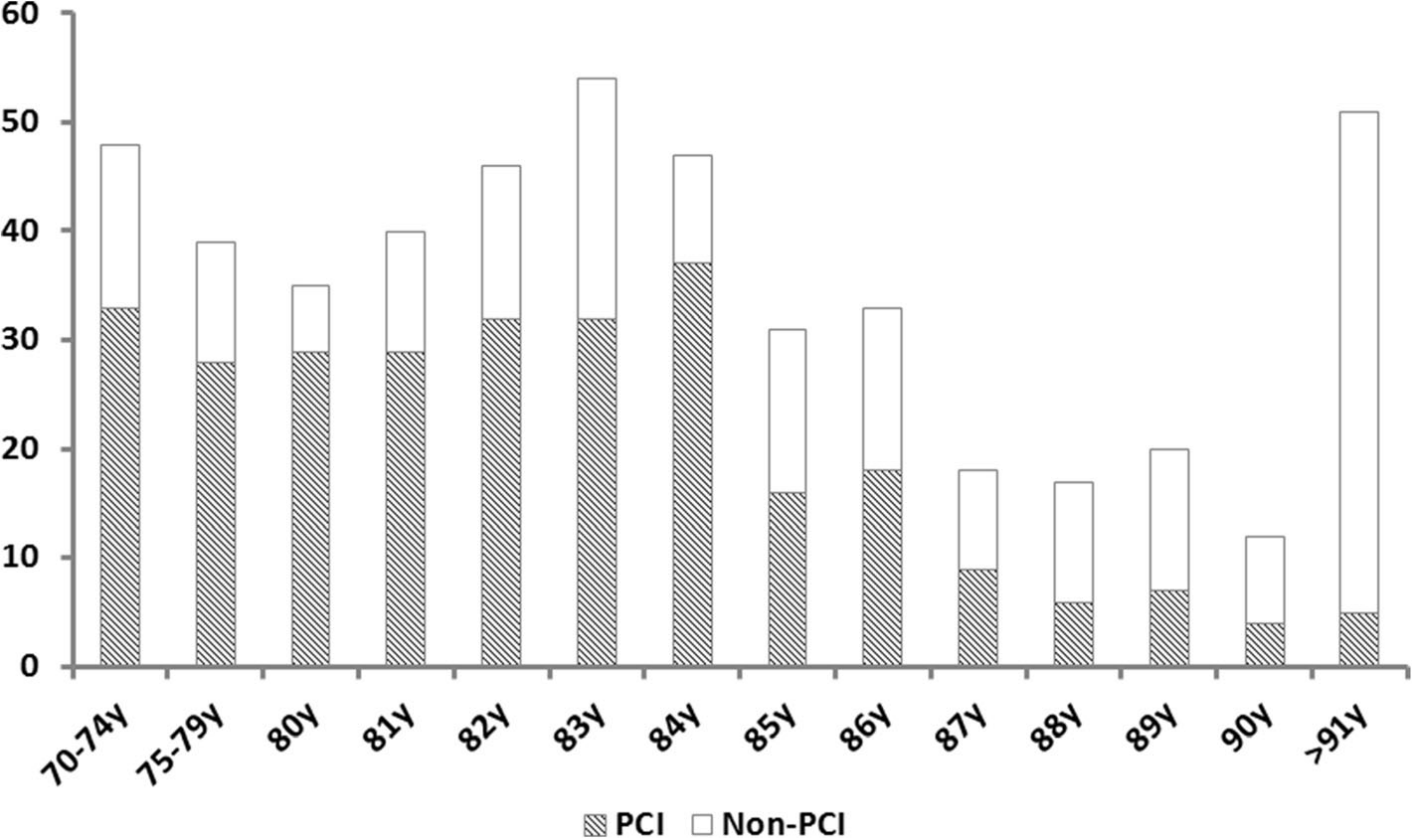
Numbers of elderly patients treated or not treated with PCI according to chronological age

The baseline characteristics are summarized in Table 1. In the overall cohort, the study population was aged around 83.2 ± 5.6 years. Among them, 285 (58%) patients had undergone PCI, whereas 206 were not. The numbers of elderly patients treated or not treated with PCI according to chronological age were illustrated in Fig. 1. In the PCI group, patients were significantly younger (81.6 ± 4.7 vs. 85.4 ± 6.0, *p* < 0.001) and were often male (66.3% vs. 46.1%, *p* < 0.001). In PCI group, STEMI, NSTEMI and UA were 54, 35 and 12%, respectively whereas 13, 79 and 8% in non-PCI group. Furthermore, in PCI group patients had higher level of hemoglobin and eGFR whereas in non-PCI group patients had more comorbidities, including history of heart failure (29.1% vs. 15.4%, *p* < 0.001), hypertension (80.5% vs. 52.3%, *p* < 0.001) and stroke (24.8% vs. 10.5%, *p* < 0.001), compared with those in PCI group. Patients undergoing PCI were less frequently treated with diuretics (28.1% vs. 49.1%, *p* < 0.001),but more frequently received statins (49.1% vs. 29.1%, *p* < 0.001) and angiotensin converting enzyme inhibitor (ACEI) or angiotensin receptor antagonist (ARB) (48.1% vs. 36.4%, *p* = 0.013).

**Fig. 2:**
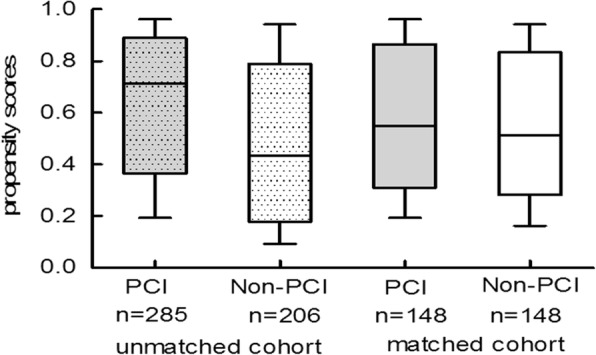
Propensity scores for treated with or without PCI in the unmatched and matched populations. Boxes represent median and interquartile range, vertical lines represent minimum and maximum

After propensity score matching, 296 patients remained including 148 in the PCI group and 148 in non-PCI group. As illustrated in Fig. 2, propensity scores were nearly identically distributed.

### Outcomes

**Fig. 3:**
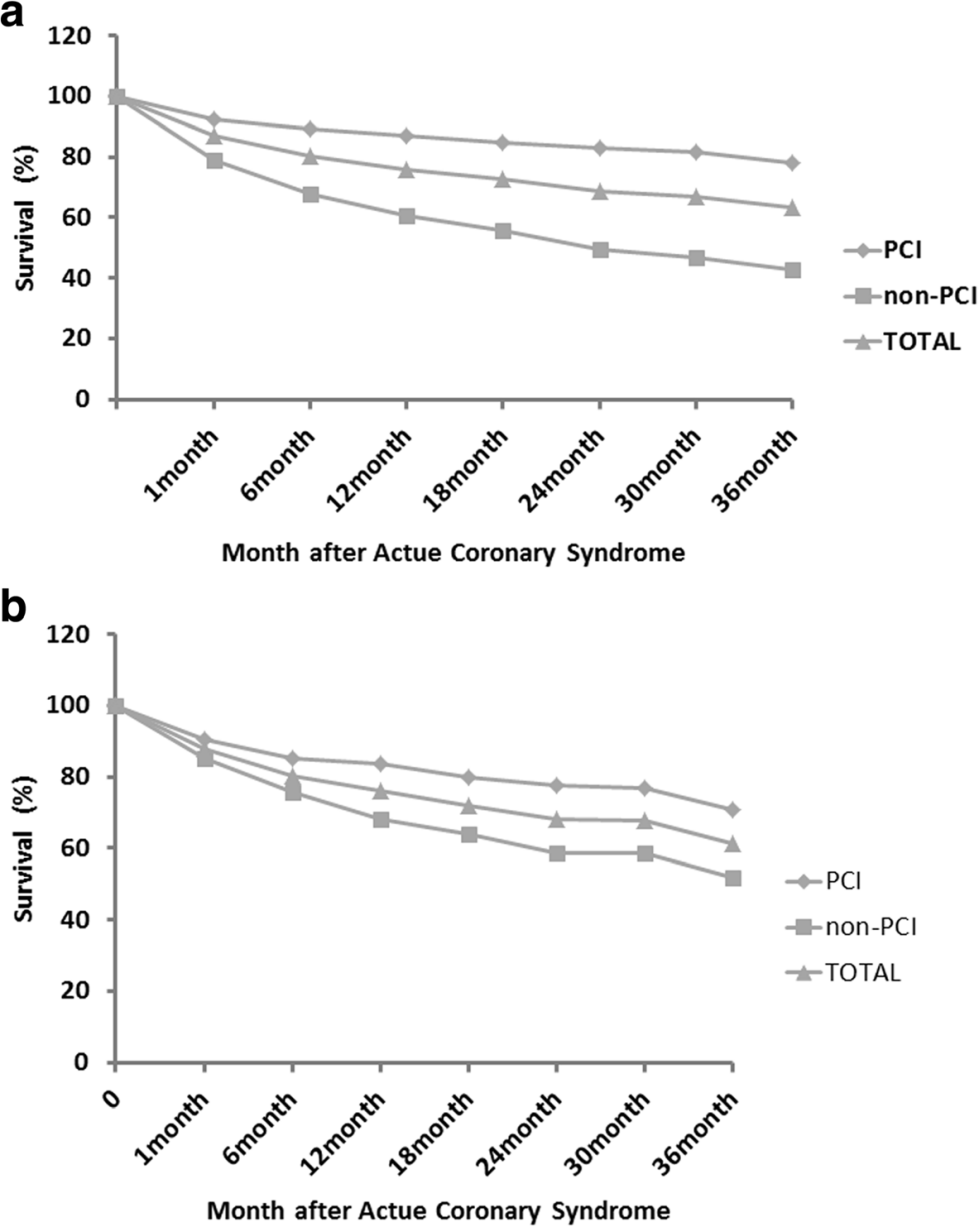
Comparison of survival of elderly patients treated with or without PCI after acute coronary syndrome. **a**: overall cohort; **b**: the matched cohort

In the overall cohort, patients in PCI group had better short and long term survival with all-cause mortality 7.4, 13, and 21.8% at 30 days, 1 and 3 years, respectively compared with 20.8, 39.3, 57.3% in non-PCI group (all *p* < 0.001). (Table 2 and Fig. 3).

**Table 2:** Association between PCI use and outcomes

Study group	Unmatched population					Matched population				
	Overall	Non-PCI (*n* = 206)	PCI (*n* = 285)	HR (95%CI)	*P*-value	Overall	Non-PCI (*n* = 148)	PCI (*n* = 148)	HR (95% CI)	*P*-value
	No events(%) IR(*100py)	No events(%) IR(*100py)	No events(%) IR(*100py)			No events(%) IR(*100py)	No events(%) IR(*100py)	No events(%) IR(*100py)		
30 days all- cause death	64 (13.0) 15.6	43 (20.8) 25.1	21 (7.4) 8.8	2.833 (1.681–4.773)	< 0.001	36 (12.2) 14.6	22 (14.9) 17.8	14 (9.5) 11.4	1.571 (0.804–3.071)	0.186
1 year all- cause death	118 (24.0) 24.0	81 (39.3) 39.3	37 (13.0) 13.0	3.393 (2.298–5.009)	< 0.001	71 (24.0) 24.0	47 (31.8) 31.8	24 (16.2) 16.2	2.062 (1.261–3.372)	0.004
3 year all- cause death	180 (36.7) 12.2	118 (57.3) 19.1	62 (21.8) 7.3	3.361 (2.468–4.578)	< 0.001	114 (38.5) 12.8	71 (48.0) 16.0	43 (29.1) 9.7	1.879 (1.286–2.745)	0.001

*IR* incidence rate, no./100 person-yr,HR, hazard ratio; *P*-value, Non-PCI vs. PCI

In the matched population, no significant difference in all-cause mortality was observed in PCI group compared to non-PCI group at 30 days (9.5% vs.14.9%, *p* = 0.186). However, patients in PCI group had better survival at 1 and 3 year (16.2 and 29.1%, *p* < 0.001) compared to non-PCI group (31.8 and 48%, *p* < 0.001) (Fig. 3).

### Prognostic predictors of 3-year mortality

On the basis of the multivariate Cox proportional-hazards model following factors were identified as independent predictor of 3-year all-cause mortality: age (HR = 1.078, 95% CI 1.001–1.162, *p* = 0.048), heart rate (HR = 1.015, 95% CI 1.005–1.026, *p* = 0.003), eGFR≤35 ml/min/1.73 m2 (HR = 3.069, 95% CI 1.633–5.770, *p* < 0.001), history of malignancy (HR = 2.334, 95%CI 1.296–4.567, *p* = 0.006), prior CABG (HR = 3.247, 95% CI 1.381–7.638, *p* = 0.007), medication with statin (HR = 0.402, 95%CI 0.188–0.862, *p* = 0.014) in PCI group, while age (HR = 1.079, 95%CI 1.028–1.132, *p* = 0.002), heart rate (HR = 1.014, 95%CI 1.005–1.023, *p* = 0.002), history of hypertension (HR = 1.872, 95%CI 1.005–3.489, *p* = 0.048) and using of ACEI/ARB (HR = 0.461, 95%CI 0.279–0.761, *p* = 0.002) in non-PCI group (Table 3).

**Table 3:** Risk factors influencing 3-year all cause mortality of patient in two groups

Variables	PCI			Non-PCI		
	HR	95%CI	*p*-value	HR	95%CI	*p*-value
Age, year	1.078	1.001–1.162	0.048	1.079	1.028–1.132	0.002
Gender, female	0.934	0.508–1.718	0.827	0.751	0.492–1.145	0.184
BMI, kg/m^2^	0.941	0.874–1.013	0.103	0.972	0.923–1.023	0.280
Smoking	1.040	0.593–1.824	0.891	0.964	0.633–1.468	0.863
Heart rate,bpm	1.015	1.005–1.026	0.003	1.014	1.005–1.022	0.002
Systolic BP, mmHg	0.996	0.986–1.006	0.404	0.990	0.983–0.997	0.007
eGFR≤35 ml/min/1.73m^2^	3.069	1.633–5.770	< 0.001	1.153	0.762–1.745	0.499
History of HF	1.261	0.665–2.391	0.478	1.338	0.866–2.068	0.190
Hypertension	1.464	0.820–2.614	0.198	1.872	1.005–3.489	0.048
Anemia	0.336	0.099–1.136	0.079	0.924	0.513–1.663	0.791
Malignancies	2.334	1.296–4.569	0.006	1.150	0.544–2.431	0.714
Stroke	0.739	0.306–1.787	0.502	1.050	0.655–1.683	0.839
Prior CABG	3.247	1.381–7.638	0.007	1.595	0.886–2.872	0.120
Prior PCI	0.639	0.242–1.686	0.366	0.779	0.362–1.677	0.524
β- Blockers	1.118	0.624–2.002	0.707	1.141	0.756–1.721	0.531
Aspirin	1.336	0.710–2.512	0.369	0.900	0.602–1.346	0.607
ACEI/ARB	1.211	0.645–2.276	0.551	0.461	0.279–0.761	0.002
Statin	0.402	0.188–0.862	0.014	0.995	0.524–1.889	0.989

### Factors associated with choice of PCI

The main determinants of choice of PCI were summarized in Table 4. Multivariable logistic regression analysis showed that age (OR 0.923, 95%CI 0.874–0.975, *p* = 0.004), male gender (OR 1.647, 95%CI 1.068–2.540, *p* = 0.024), lower heart rate (OR 0.900, 95%CI 0.981–0.999, *p* = 0.032), lower systolic blood pressure (OR 1.009, 95%CI 1.001–01.017, *p* = 0.030), without history of hypertension (OR 2.699, 95%CI 1.689–4.313, *p* < 0.001) and stroke (OR 2.425, 95%CI 1.389–4.233, *p* = 0.002) were the independent factors in favour of choice of PCI.

**Table 4:** Baseline characteristics related to the propensity using of PCI

Variables	Univariable			Multivariable		
	OR	(95%CI)	p	OR	(95%CI)	p
Age, year	0.870	0.837–0.905	< 0.001	0.923	0.874–0.975	0.004
Gender, female	2.300	1.592–3.323	< 0.001	1.647	1.068–2.540	0.024
Heart rate,bpm	0.986	0.977–0.994	0.001	0.900	0.981–0.999	0.032
Systolic BP, mmHg	1.007	1.000–1.013	0.055	1.009	1.001–1.017	0.030
eGFR, ml/min/1.73m^2^	1.026	1.016–1.036	< 0.001	1.005	0.993–1.018	0.393
History of HF	2.251	1.450–3.945	< 0.001	1.142	0.666–1.957	0.630
Hypertension	3.788	2.498–5.743	< 0.001	2.699	1.689–4.313	< 0.001
Stroke	2.797	1.708–4.580	< 0.001	2.425	1.389–4.233	0.002
Malignancies	0.543	0.289–1.021	0.058	0.512	0.250–1.046	0.066
Prior CABG	1.576	0.858–2.897	0.143	1.847	0.873–3.908	0.109
Prior PCI	0.928	0.529–1.628	0.794	1.278	0.650–2.513	0.478
ACEI/ARB	0.618	0.429–0.893	0.010	1.006	0.619–1.635	0.980
Diuretic	2.465	1.692–3.590	< 0.001	1.489	0.936–2.368	0.093
Statin	0.420	0.287–0.614	< 0.001	0.725	0.423–1.242	0.242

## Discussions

Our results demonstrated that PCI was associated with better survival in both short and long terms in an elderly cohort with ACS. Moreover, the prognostic factors of 3-year all-cause mortality between PCI and non-PCI group are different. Finally, the decision to proceed with PCI in the elderly ACS population was affected by the age, gender, blood pressure, heart rate, hypertension and stroke.

Older patients are often accompanied by more comorbidity and more spread atherosclerotic disease, PCI is therefore more challenging from the technical point of view. Recently, there were data indicating that PCI in elderly seems to be associated with good early and intermediate outcomes [11, 16]. Our results are in line with previous findings by showing that the elderly ACS patients can get benefit of PCI not only at 1-year but also 3-years. Recently a randomised study aimed to investigate whether elderly patients with NSTEMI or unstable angina would benefit from an early invasive strategy versus a conservative strategy and showed similar results as ours [14]. Although the mean age (85.4 years in non-PCI group and 81.6 years in PCI group) and sample size (*n* = 491) in our study are comparable to above-mentioned randomized study (84.7 in invasive strategy group and 84.9 in conservative strategy group) (*n* = 457), there are several differences: First, as inclusion, no patients with STEMI was included in randomized study. However STEMI is a large proportions of the total ACS patients included in our study. Second, as many other trials randomized study was subject to many exclusions such as cardiogenic shock, continuing bleeding problems, short life expectancy (such as chronic obstructive pulmonary disease, disseminated malignant disease, or others), and substantial mental disorder. This was not the case in our study. Therefore our study is more close to the “real world” of the clinical practice. Third, the invasive strategy in the randomized study included early coronary angiography with PCI and CABG, but in our study we assessed only association between PCI and the outcomes. Most important, above randomized study was underpowered to assess survival. Last, like most clinical trials and retrospective studies involving elderly patients they seldom have long-term results and the median follow-up in the randomized study is only 1.53 years.

Our data about 30-day, 1 and 3-year mortality are in line with the previous studies in elderly patients in whom 30 day mortality was reported to be 7 to 15% [17–19], and 15 to 21% of patients treated with conservative strategy [20, 21]. In our study, the 1-year mortality rate in octogenarian population a is consistent with the German ALKK registry [16] in which all-cause mortality in patient 65–74 years was 10.1, and 20.4% in patients≥75 undergoing PCI [22], and upon 1 year follow-up the mortality rate among the invasive group was lower compared with medically managed patients (11–20% vs. 19–30%) [23–26]. Long term data are relatively sparse, our 3-year mortality rates for elderly were comparable to previous studies in which 3-year all cause death in elderly patients with mean age of 88 years old treated with PCI were 33.3%, and treated with conservative management were 52.4% [27]. Taken all results together, our study extended previous observations by showing that PCI was associated with better survival in both short- and long- terms in an elderly cohort with ACS.

Previous studies showed that preoperative factors influencing mortality in elderly treated with invasive or conservative strategy are cardiogenic shock, previous cardiac surgery, renal failure, age, hypertension, heart rate and anemia [24, 28–33]. Moreover it is known that medication use during PCI is related to success and less complications, for example, statin use may reduce complications and mortality after PCI [34], which was confirmed in this study. We find that age, heart rate, eGFR, malignance, history of prior CABG, using of statin were independent predictor of 3-year all-cause mortality in PCI group, whereas age, heart rate, systolic BP and using of ACEI/ARB were independent predictor of 3-year all-cause mortality in non-PCI group.

The decision to proceed with a PCI procedure in the elderly is influenced by numerous factors. Non-cardiac comorbidities include renal, cerebral, pulmonary and vascular disease [35] were often taken into account prior to decision making for PCI. There was a tendency to select intervention management in patients with a lower risk profile [1, 22, 36]. This is also reflected in our cohort of elderly patients. Although we cannot tell what factors led to these patients being selected for PCI, our study confirmed that the determinants for choosing PCI included younger age, male gender, lower heart rate, lower systolic BP, without history of hypertension and stroke.

### Limitations and strengths

Despite our efforts in catching up as much information as possible in medical records, and despite PS adjustment, we cannot rule out potential confounding from unmeasured variables. Nevertheless, our data are encouraging and serve as the basis for randomized trials in the future. One of the main strengths is that all patients were included from our daily clinical practice, and therefore representative.

## Conclusion

In elderly ACS patients, PCI-treatment was associated with improved 1 and 3-year survival and PCI-treated patients had different prognostic profile compared to those without PCI treatment.

## Abbreviations

ACEI: angiotensin converting enzyme inhibitor
ACS: acute coronary syndromes
ARB: angiotensin receptor antagonist
BP: blood pressure
CABG: coronary artery bypass grafting
NSTEMI: non-ST segement elevation myocardial infarction
PCI: percutaneous coronary intervention
STEMI: ST segment elevation myocardial infarction
UA: unstable angina

## References

[1] Avezum A, Makdisse M, Spencer F, Gore JM, Fox KA, Montalescot G (2005). Impact of age on management and outcome of acute coronary syndrome: observations from the global registry of acute coronary events (GRACE). Am Heart J.

[2] Alexander KP, Newby LK, Armstrong PW, Cannon CP, Gibler WB, Rich MW (2007). Acute coronary care in the elderly, part II: ST-segment-elevation myocardial infarction: a scientific statement for healthcare professionals from the American Heart Association Council on clinical cardiology: in collaboration with the Society of Geriatric Cardiology. Circulation.

[3] Alexander KP, Newby LK, Cannon CP, Armstrong PW, Gibler WB, Rich MW (2007). Acute coronary care in the elderly, part I: non-ST-segment-elevation acute coronary syndromes: a scientific statement for healthcare professionals from the American Heart Association Council on clinical cardiology: in collaboration with the Society of Geriatric Cardiology. Circulation.

[4] Force m A/T, Windecker S, Kolh P, Alfonso F, Collet JP, Cremer J (2014). 2014 ESC/EACTS guidelines on myocardial revascularization: the task force on myocardial revascularization of the European Society of Cardiology (ESC) and the European Association for Cardio-Thoracic Surgery (EACTS)developed with the special contribution of the European Association of Percutaneous Cardiovascular Interventions (EAPCI). Eur Heart J.

[5] Dzavik V, Sleeper LA, Cocke TP, Moscucci M, Saucedo J, Hosat S (2003). Early revascularization is associated with improved survival in elderly patients with acute myocardial infarction complicated by cardiogenic shock: a report from the SHOCK trial registry. Eur Heart J.

[6] Dauerman HL, Bhatt DL, Gretler DD, French PA, Smyth SS, Becker RC (2010). Bridging the gap between clinical trials of antiplatelet therapies and applications among elderly patients. Am Heart J.

[7] Graham MM, Ghali WA, Faris PD, Norris CM, Knudtson ML (2002). Alberta provincial project for outcomes assessment in coronary heart disease(APPROACH) investigators. Survival after coronary revascularization in the elderly. Circulation.

[8] Malyszko J, Bachorzewska-Gajewska H, Malyszko JS, Dobrzycki S (2010). Prevalence of chronic kidney disease in elderly patients with normal serum creatinine levels undergoing percutaneous coronary interventions. Gerontology.

[9] Radovanovic D, Urban P, Simon R, Schmidli M, Maggiorini M, Rickli H (2010). Outcome of patients with acute coronary syndrome in hospitals of different sizes. A report from the AMIS plus registry. Swiss Med Wkly.

[10] Devlin G, Gore JM, Elliott J, Wijesinghe N, Eagle KA, Avezum A (2008). Management and 6-month outcomes in elderly and very elderly patients with high-risk non-ST-elevation acute coronary syndromes: the global registry of acute coronary events. Eur Heart J.

[11] McKellar SH, Brown ML, Frye RL, Schaff HV, Sundt TM (2008). Comparison of coronary revascularization procedures in octogenarians: a systematic review and meta-analysis. Nat Clin Pract Cardiovasc Med.

[12] de Boer SP, Westerhout CM, Simes RJ, Granger CB, Zijlstra F, Boersma E (2010). Mortality and morbidity reduction by primary percutaneous coronary intervention is independent of the patient's age. JACC Cardiovasc Interv.

[13] Barywani SB, Li S, Lindh M, Ekelund J, Petzold M, Albertsson P (2015). Acute coronary syndrome in octogenarians: association between percutaneous coronary intervention and long-term mortality. Clin Interv Aging.

[14] Tegn N, Abdelnoor M, Aaberge L, Endresen K, Smith P, Aakhus S (2016). Invasive versus conservative strategy in patients aged 80 years or older with non-ST-elevation myocardial infarction or unstable angina pectoris (After Eighty study): an open-label randomised controlled trial. Lancet.

[15] D’ Agostino RB (1998). Propensity score methods for bias reduction in the comparison of a treatment to a non-randomized control group. Stat Med.

[16] Rittger H, Hochadel M, Behrens S, Hauptmann KE, Zahn R, Mudra H (2012). Age-related differences in diagnosis, treatment and outcome of acute coronary syndromes: results from the German ALKK registry. Euro Intervention.

[17] Jaguszewski M, Ghadri JR, Diekmann J, Bataiose RD, Hellermann JP, Sarcon A (2015). Acute coronary syndromes in octogenarians referred for invasive evaluation: treatment profile and outcomes. Clin Res Cardiol.

[18] de Boer MJ, Ottervanger JP, Suryapranata H, Hoorntje JC, Dambrink JH, Gosselink AT (2010). Old age and outcome after primary angioplasty for acute myocardial infarction. J Am Geriatr Soc.

[19] Moreno R, Salazar A, Banuelos C, Hernadez R, Alfonso F, Sabate M (2004). Effectiveness of percutaneous coronary interventions in nonagenarians. Am J Cardiol.

[20] Vassalli G, d'Angeli I, Scherff F, Surder D, Mantovani A, Pasotti E (2015). Comparison of clinical and angiographic prognostic risk scores in elderly patients presenting with acute coronary syndrome and referred for percutaneous coronary intervention. Swiss Med Wkly.

[21] Rosengren A, Wallentin L, Simoons M, Gitt AK, Behar S, Battler A (2006). Age, clinical presentation, and outcome of acute coronary syndromes in the Euroheart acute coronary syndrome survey. Eur Heart J.

[22] Yan RT, Yan AT, Tan M, Chow CM, Fitchett DH, Ervin FL (2006). Age-related differences in the management and outcome of patients with acute coronary syndromes. Am Heart J.

[23] Antonsen L, Jensen LO, Terkelsen CJ, Tilsted HH, Junker A, Maeng M (2013). Outcomes after primary percutaneous coronary intervention in octogenarians and nonagenarians with ST-segment elevation myocardial infarction: from the western Denmark heart registry. Catheter Cardiovasc Interv.

[24] Gunal A, Aengevaeren WR, Gehlmann HR, Luijten JE, Bos JS, Verheugt FW (2008). Outcome and quality of life one year after percutaneous coronary interventions in octogenarians. Neth Heart J.

[25] Guagliumi G, Stone GW, Cox DA, Stuckey T, Tcheng JE, Turco M, Musumeci G (2004). Outcome in elderly patients undergoing primary coronary intervention for acute myocardial infarction: results from the controlled Abciximab and device investigation to lower late angioplasty complications (CADILLAC) trial. Circulation 21.

[26] Bauer T, Koeth O, Junger C, Heer T, Wienbergen H, Gitt A (2007). Effect of an invasive strategy on in-hospital outcome in elderly patients with non-ST-elevation myocardial infarction. Eur Heart J.

[27] Munoz JC, Alonso JJ, Duran JM, Gimeno F, Ramons B, Garcimatin I (2002). Coronary stent implantation in patients older than 75 years of age: clinical profile and initial and long-term (3 years) outcome. Am Heart J.

[28] Batchelor WB, Anstrom KJ, Muhlbaier LH, Grosswald R, Weintraub WS, O’Neill WW (2000). Contemporary outcome trends in the elderly undergoing percutaneous coronary interventions: results in 7,472 octogenarians. National Cardiovascular Network Collaboration. J Am Coll Cardiol.

[29] Weintraub WS, Veledar E, Thompson T, Burnette J, Jurkovitz C, Mahoney E (2001). Percutaneous coronary intervention outcomes in octogenarians during the stent era (National Cardiovascular Network). Am J Cardiol.

[30] Yayan J (2014). Association of traditional risk factors with coronary artery disease in nonagenarians: the primary role of hypertension. Clin Interv Aging.

[31] Granger CB, Goldberg RJ, Dabbous O, Pieper KS, Eagle KA, Cannon CP (2003). Predictors of hospital mortality in the global registry of acute coronary events. Arch Intern Med.

[32] Jensen MT, Pereira M, Araujo C, Malmivaara A, Ferrieres J, Degano IR, et al. Heart rate at admission is a predictor of in-hospital mortality in patients with acute coronary syndromes: results from 58 European hospitals: the European hospital benchmarking by outcomes in acute coronary syndrome processes study. Eur Heart J Acute Cardiovasc Care. 2016; 10.1177/2048872616672077 27694532

[33] Cavusoglu E, Chopra V, Gupta A, Clark LT, Eng C, Marmur JD (2006). Usefulness of anemia in men as an independent predictor of two-year cardiovascular outcome in patients presenting with acute coronary syndrome. Am J Cardiol.

[34] Herrmann J, Lerman A, Baumgart D, Volbracht L, Schulz R, von Birgelenc C (2002). Preprocedural statin medication reduces the extent of periprocedural non-Q-wave myocardial infarction. Circulation.

[35] Klein LW, Block P, Brindis RG, McKay CR, McCallister BD, Wolk M (2002). Percutaneous coronary interventions in octogenarians in the American College of Cardiology-National Cardiovascular Data Registry: development of a nomogram predictive of in-hospital mortality. J Am Coll Cardiol.

[36] Bhatt DL, Roe MT, Peterson ED, Li Y, Chen AY, Harrigton RA (2004). Utilization of early invasive management strategies for high-risk patients with non-ST-segment elevation acute coronary syndromes: results from the CRUSADE quality improvement initiative. JAMA.

